# Contextual Exceptionalism After Death: An Information Ethics Approach to Post-Mortem Privacy in Health Data Research

**DOI:** 10.1007/s11948-022-00387-0

**Published:** 2022-08-03

**Authors:** Marieke A. R. Bak, Dick L. Willems

**Affiliations:** Department of Ethics, Law and Humanities, Amsterdam UMC (Location AMC), University of Amsterdam, Meibergdreef 9, 1105 AZ Amsterdam, The Netherlands

**Keywords:** Death, Health data, Privacy, Research ethics, Genetics, Philosophy of information

## Abstract

In this article, we use the theory of Information Ethics to argue that deceased people have a *prima facie* moral right to privacy in the context of health data research, and that this should be reflected in regulation and guidelines. After death, people are no longer biological subjects but continue to exist as informational entities which can still be harmed/damaged. We find that while the instrumental value of recognising post-mortem privacy lies in the preservation of the social contract for health research, its intrinsic value is grounded in respect for the dignity of the post-mortem informational entity. However, existing guidance on post-mortem data protection is available only in the context of genetic studies. In comparing the characteristics of genetic data and other health-related data, we identify two features of DNA often given as arguments for this genetic exceptionalism: relationality and embodiment. We use these concepts to show that at the appropriate Level of Abstraction, there is no morally relevant distinction between posthumous genetic and other health data. Thus, genetic data should not automatically receive special moral status after death. Instead we make a plea for ‘contextual exceptionalism’. Our analysis concludes by reflecting on a real-world case and providing suggestions for contextual factors that researchers and oversight bodies should take into account when designing and evaluating research projects with health data from deceased subjects.

## Introduction

Deceased people are making a growing contribution to the pool of personal data used for health research. The rise of longitudinal studies and long-term biobanking projects increases the proportion of research participants who have since passed away, especially for diseases with high mortality risks. In addition, many clinical data used for research relate to deceased people who were not participants in a study during their lifetime, e.g. people who died from acute illness (Bak et al., 2018). However, in Europe the General Data Protection Regulation (GDPR) does not directly apply to deceased persons, causing Member States to vary in their enactment of provisions regarding dead data subjects.^1^ Post-mortem data use is generally not discussed in study consent forms and few international^2^ guidelines address this topic (Harbinja, 2019; Hirschberg et al., 2013; Jurate et al., 2014; Tassé, 2011).

In the near future, general data donation schemes may enable people to donate all their personal data after death, similar to donating organs or bodies (Jones et al., 2017; Krutzinna et al., 2019a; Shaw et al., 2015). This already exists for certain datasets, such as full genomes donated through the Personal Genome Project. The draft of “an ethical code for posthumous medical data donation [PMDD]” is also available (Krutzinna et al., 2019b). For the purpose of this paper, however, we do not discuss PMDD registries but focus on data collected for specific research projects, because that is currently still the largest source of posthumous research data. Present-day regulations fall short in this context and clinicians are often unsure whether they can use data for research when the patient is dead. This can lead to arbitrary decisions, producing undesirable consequences for individuals and society if data are used without appropriate governance, or not used at all (Jansen et al., 2007; Sorbie, 2019).

The guidance that does exist (e.g. Laurie, 2004) focuses on information contained in *genetic samples*, as does ethics oversight. Scholarly literature similarly focuses on genetics rather than on other health data (e.g., pharmaceutical records or imaging data) (Bak et al., 2020). For genetic data, it seems commonly accepted that they come with at least some post-mortem privacy rights, but for other data there is almost no guidance. Elsewhere, we have noted that the focus on genetic data “obscures the question whether research participants should have post-mortem informational self-determination as such, independent of whether their data have health implications for their relatives” (ibid). In this paper, we provide a conceptual analysis of post-mortem privacy grounded in Floridian information ethics (Floridi, 2013). Whether guidance for researchers should lean towards individual control or towards solidarity and ‘presumed’ consent, or sit somewhere in the middle, depends on a number of philosophical and practical assertions. Therefore, we investigate the following questions:

Q1.Is post-mortem harm to participants in health data research possible, and if so how?

Q2. What is the nature of post-mortem genetic and other health data and is there a substantive moral difference between the two?

Q3. What guidelines follow from this for researchers and oversight bodies?

The rest of the article is structured in four sections. Section 2 sketches the philosophical debate on posthumous harm. Section 3 asserts that deceased research subjects’ privacy can still be violated (Q1), and Sect. 4 argues that there is no essential difference between genetic and other health-related data in terms of the potential for harm (Q2). Section 5 provides recommendations for practice and further study (Q3).

## Background on Posthumous Harm

Most people agree that acts like defaming someone posthumously, or not fulfilling a deathbed promise, are morally wrong. Is this because the dead can be harmed, or merely because doing so violates the virtue of respecting others? Hereafter we sketch the historical debate on the possibility of post-mortem harm,^3^ which is relevant for our topic as breaches of data privacy are essentially harms, that is, setbacks to someone’s interest in their personal identity (Floridi, 2005). Belliotti (2011) proposes that two criteria are at the core of the debate: the *experience requirement* (i.e., the subject of the harm should experience the sensations that belong to being harmed) and the *existence requirement* (i.e., the subject of the harm should have interests, that is, be living and identifiable).

### The Experience Requirement Revisited

Post-mortem harm was already debated among ancient philosophers. In the *Nicomachean Ethics*, Aristotle suggests that concerns for reputation and for the well-being of offspring transcend people’s lifetimes (Aristotle, 1953).^4^ This belief in post-mortem harm, albeit weak, is in contrast with Epicurus’s well-known annihilation thesis: the idea that since death is the end of sensations, posthumous harm cannot be experienced and hence does not occur:

…death means nothing to us, since every good and every evil lies in sensation; but death is the privation of sensation. (…) So long as we are existent death is not present and whenever it is present we are non-existent. Thus it is of no concern either to the living or to those who have completed their lives. (Epicurus, 2012)

Epicurus used the annihilation thesis primarily to refute death anxiety. Yet contemporary thinkers like Partridge and Taylor also say that harm occurs only when the person is ‘worse off’ as measured by his or her own subjective account (Partridge, 1981; Taylor, 2005). They think there is no such thing as unaffected harm: while a posthumous act like defamation may be *wrong*, defaming does not *harm* the dead. Other modern-day philosophers convincingly reject this experience requirement by highlighting the difference between harm and hurt. Nagel (1970) argues that harming someone is bad not because the victim would be upset when discovering the harm, but because an independent observer would recognize that it is bad to be the (unaware) victim. We have an interest in privacy as such, not merely in believing our privacy is respected. In a similarly Aristotelian essay, Feinberg (1974) states that harm can be objectively evaluated, and that interests can still be affected when they can no longer be experienced by the interest-holder. The dead can be harmed even though they cannot be hurt (Feinberg, 1977; Parfit, 1984). Whether post-mortem data research without consent would actually constitute harm to the deceased patient, will be discussed in Sect. 3. For now, the point is that someone can be harmed without their knowledge, i.e. the experience requirement is deemed false.

Of note is that opponents and proponents of the experience requirement agree on the societal value of the recognition of post-mortem harm by surviving parties. Part of the human condition is that we all have “transcendent interests” (Belliotti, 2011) that persist after death, such as care for our children or community, or how our corpse is handled. Disrespecting the rights that correspond with these interests harms the social contract between living people. For instance, neglecting people’s wills would adversely affect our trust in others (Partridge, 1981; see also Glannon, 2001). The continuation of the social contract requires people to act at least *as if* there is such a thing as post-mortem harm, or what Partridge calls ‘quasi-harms’. This is true even when no-one is around to experience the harm.

### The Problem of the Missing Patient

It would be too hasty to move from the premise that hurt is not required for harm, to the conclusion that the dead can be harmed. Namely, the existence requirement is more difficult to refute because it is hard to establish who bears the interests of the deceased: this is known as ‘the problem of the missing subject’ (Waluchow, 1986). Some have suggested that to be the relatives, but not all people have surviving relatives. Or that those who outlive the deceased person, be that their relatives or members of the community, bear the duty of fulfilling the interests of the deceased person or even of a timeless ‘human subject’ as symbolic rights-holder (Belliotti, 2011; Scarre, 2003; Sperling, 2008). These suggestions go well with the social contract position but are not true accounts of post-mortem harm.

Another suggestion stems from Kant, who recognised that even if we cease to exist after death, our reputation lives on (Kant, 2017). Breaching a deceased patient’s privacy would be bad because it involves treating the patient as a mere means to the researcher’s ends. The subject of the harm after death is the *living* person who had an interest in privacy; the *ante-mortem* subject ‘who is robbed of their honour as if it happened during their lifetime’, to paraphrase Kant. Following this reasoning, Feinberg and Pitcher have argued that harm begins when the victim first develops the associated interest, i.e. the patient’s privacy was violated ‘all along’ by the post-mortem breach. However, this view is difficult to accept as it does not align with common intuitions and with ideas about responsibility (was the perpetrator then responsible already before the harm occurred?).

If we take the existence requirement to mean simply that there should be an existing subject, then the dead *themselves* are the appropriate interest-holders: they continue to exist as fact (‘reputational’) information about persons from the past (Glannon, 2001; Tandy, 2003). Consider the sentence “Henrietta Lacks’s cells have made a major contribution to medicine”. This true fact is caused by the existence of Henrietta Lacks; which means she must continue to exist not only biologically in the form of HeLa cells, but also as *informational entity* (cf. the example of Socrates in Catterson, 2003).^5^ Under this informational view of existence we could also harm inanimate objects. To avoid that conclusion, the existence requirement is often interpreted as an ‘aliveness requirement’ which leads to circular reasoning: the dead cannot be harmed because harm requires a living and sentient being. But why could someone in a persistent vegetative state be harmed, and not a dead person? Both individuals’ lives would have been “more successful if the interests they formed while alive and conscious flourish when they are unconscious or dead” (Dworkin, 2008).

Therefore we argue, in line with Nagel, that a living subject is indeed required for being physically or psychologically *hurt* but that dead people can still be harmed. In the next section, we discuss the implications of this view for post-mortem privacy in health data research.

## Conceptualising Post-Mortem Privacy in Health Data Research

Posthumous data research gives patients the opportunity to reciprocate: some authors have advocated a human right to participate in science (Vayena & Tasioulas, 2015). In our own empirical research, survivors of sudden cardiac arrest stated that “when you are dead, you are dead, but it would be nice if you can prevent other people from dying in the same way” (Bak et al., 2021). Nonetheless, research with the deceased has always been an ethically sensitive topic, as controversies have shown, for example, relating to the publication of Vincent van Gogh’s medical record, tests performed on Albert Einstein’s brain, or the excavation of remains from people in disadvantaged tribal communities (Ploem, Bak, Linthorst 2021; Nelkin & Andrews, 1998). In those cases, innovative techniques enabled previously impossible research, while the dead have no opportunity to control these new uses.

In a study among internet users in Israel, half of respondents wished to limit access to their digital remains (Morse & Birnhack, 2020). In the context of medical research, most stakeholders think that participants should be informed about post-mortem data uses while they are still alive (Bak et al., 2020). Proper handling of post-mortem data can promote public trust in researchers, while a lack of consent from either patient or relatives may lead to mistrust in the research enterprise. When (intangible) expectations on posthumous data use are disrespected, this collapse of context could lead to reluctance to participate, resembling the UK’s failed *care.data* scheme (Vezyridis & Timmons, 2017). The care.data programme aimed to develop a national database of hospital and general practice records, in collaboration with commercial partners, but was cancelled after backlash from the public who had not been properly informed about the uses of their data. Such public distrust hampers medical research by limiting the available data and by creating bias in datasets when people with similar characteristics (e.g., in terms of age group or personality type (Skatova & Goulding, 2019)) refuse to give their data (Jones et al., 2017; Tu et al., 2004).

Thus, the *instrumental* value of protecting post-mortem privacy lies in safeguarding the social contract for health data research, but what about the *intrinsic* value of recognising deceased subjects’ privacy? We analyse the latter using Luciano Floridi’s Information Ethics (IE).

### An Information Ethics Approach

IE is a patient-oriented ethics that differs from bioethics because its main focus (or its main Level of Abstraction) is not on organic life, but on information as the key building block of moral patients. As such, IE builds on Floridi’s philosophy of information (PI), a family of phenomenological theories whose main thesis is that *reality is best interpreted as the totality of information*. Given the right level of abstraction (LoA), anything, from an enzyme to a healthcare system, can be presented as informational system. In the information society which is making the mental world increasingly part of the environment, humans are conscious ‘inforgs’ (i.e., informational organisms) who can create knowledge from semantic information, the latter meaning “well-formed, meaningful and truthful data” (Floridi, 2011, p. 31). In other words, data needs to be invested with meaning in order to produce *useful* information that can be used to model the world with words, numbers, and figures. In Kantian terms, data are noumena while information belongs to the realm of the phenomena (ibid, p. 40).^6^

According to IE, not only the biosphere, but also the *infosphere* can be harmed/damaged (and benefited/enhanced). Damage is defined as the change which degrades an object, animated or not, from its original state. Limbaugh (2019) states, in a different context, that “the point is not that the organism is worse off than it would have been, but rather that the organism is simply not as it should be”. Similarly, damaging information is bad because it lacks respect for the essence of informational entities.^7^ This is in line with a minimalist interpretation of the existence requirement, that is, informational entities exist and can therefore be harmed. However, as harm is often associated with living persons, damage may be a more appropriate term for cases of harm (including privacy breaches) where the biocentric ‘aliveness requirement’ is not satisfied; better than Partridge’s concept of *quasi*-harm which suggests there is *not really* anything wrong.

The concept of post-mortem data privacy can thus be formulated in terms of a moral right to be protected from damage to the “packet of information” that is the individual, and this right is grounded in respect for the *dignity* of the informational entity (Floridi, 2016; Öhman & Floridi, 2017). For instance, Stokes (2015) argues that because digital online remains persist as ethical patients, there exists a moral obligation not to delete social media profiles of deceased persons (unless that person wanted it to be deleted). The European legal framework similarly regards data as inalienable possessions, and EU laws are based on human rights such as dignity, autonomy, and respect for persons (Harbinja, 2019). When someone donates their body to science, researchers have a duty to treat the body with respect and not to damage it more than necessary; despite the consent from the ante-mortem person. The same goes for a person’s data.

While researchers have a *prima facie* moral duty to respect the dignity of data subjects, including after death, this does not mean that the interests of the dead are absolute: they may be overridden by more pressing moral considerations. There is a difference between arguing that data can always be used without consent because the dead cannot be harmed; or that data can be used without consent in specific situations where the interests of the living override those of the dead (we advocate the latter).^8^ Deceased patients, their relatives, and the broader moral community deserve different levels of moral respect that need to be weighed against each other. This can only be done in specific situations, as competing versions of dignity may be simultaneously at stake: it is health data researchers’ engagement with these dilemmas itself that is needed for respecting patients’ dignity (Pols et al., 2018). This contextual aspect is discussed in Sect. 4 where we compare the harm from post-mortem uses of genetic data with other types of health-related data.

## How Does Genetic Data Compare to Other Health Data after Death?

We have argued that recognising post-mortem privacy is important for the social contract and that dead patients can be harmed *qua* informational entities, but the question whether the harm is more serious in genetic studies than in other health data research has not received scholarly attention.^9^ This question is important because research policies and guidelines seem committed to a kind of *genetic exceptionalism* (GE).This is the belief that genetic data has an exceptional status and requires special protection (Annas et al., 1995; Murray, 1997). While the GDPR does not discriminate between genetic and other health-related data, under Article 9(4) the regulation does allow Member States to impose further limitations specifically for genetic data (Mitchell et al., 2020). In most ethics guidelines, the processing of deceased persons’ data is addressed only in the context of genetics, if they discuss post-mortem data research at all (Bak et al., 2020). In what follows we critically analyse, and compare with other health data, the two features of post-mortem genetic data that adherents of GE most frequently use for their argumentation: *relationality* and *embodiment*.

### Relationality

GE was born in the 1990s along with the Human Genome Project and ideas about genetic determinism (McGuire et al., 2008; Murray, 2019). Regardless of technical privacy concerns about re-identification of the deceased participant (and possibly their relatives),^10^ post-mortem GE may persist because deeper socio-cultural ideas underlie the notion of genetic data representing something special about the dead. Namely, with the advent of molecular biology, many complex diseases were claimed to be reducible to our genetic material (Sarkar, 1998). It was said that ‘we are our DNA’ (Nelkin & Lindee, 1995). This idea has been corroborated by commercial companies’ persuasive marketing of genetic testing, and by the limited public understanding of genetics and genomics (Schaper & Schicktanz., 2018). This understanding, and therefore the social construction of DNA, is shaped by the fact that healthcare providers and researchers communicate primarily through metaphors (e.g., the genome as a book or a hard drive consisting of all the information about who we are) (Richards, 2001).

While these factors may explain the advent of GE, they do not justify it. Most conditions are still diagnosed by physical examination rather than through genetic testing, and hereditary illness may present through phenotype and/or family history. Increasingly, non-genetic biomarkers are found that predict the risk of disease, such as the blood-based assessment of amyloid or tau pathology in Alzheimer’s disease (Schindler & Bateman, 2021). Even non-health data are used to make inferences about health (Price & Cohen, 2019). For instance, analysis of keyboard typing behaviour can help detect depressive tendencies and even predict progression of multiple sclerosis symptoms among smartphone users (Lam et al., 2021; Mastoras et al., 2019). Thus, we argue that instead of being our DNA, ‘we are our information’. The information encoded in DNA would still exist if scientists had not unravelled the genetic code, like a piece of music still exists when one plays it without sheet music.

Still, proponents of GE argue that genetic data are different because DNA analysis allows for a *definitive* diagnosis. This is not necessarily true, as genotype penetrance can differ widely. Some gene variants have little significance and their impact depends on medical or environmental factors, such as health-related behaviour (Evans & Burke, 2008). Even DNA molecules themselves are influenced by epi-genetic factors from the environment (Gräff & Mansuy, 2008). An example of a definitive diagnosis without DNA is a study on familial screening after sudden cardiac death, where “complex genetic inheritance can limit the yield of such [genetic] screening” and the authors show that clinical screening (review of medical history, physical examination, and ECG analysis), supplemented with exercise and pharmacological tests, is effective to diagnose relatives of the deceased (Quenin et al., 2017). Diagnosis was confirmed using data about the circumstances of the death.

The field of genetics is often said to be special because it may have implications for living blood relatives who share part of the deceased person’s genetic code. Relatives may not want to know about genetic findings potentially affecting their own health, and disclosure may result in psychosocial harm (Featherstone et al., 2006; Rolland, 2006). But as the example of sudden cardiac death shows, this relationality^11^ is not an exclusive feature of genetics. Other examples are medical imaging which can generate incidental findings relevant for family members, thus impacting insurance eligibility and premiums, and the results of an HIV test which can predict future health problems among sexual partners (Green & Botkin, 2003). Psychological and psychiatric data are also instances of information that is valuable for care and research but can have substantial relational privacy implications, e.g. when qualitative data contain descriptions of interpersonal traumatic events involving other people (Stein et al., 2022). Thus, we find that relationality is no valid argument for (post-mortem) GE.

We also note that while discussions about the relational component of data often focus on the deceased person’s *blood* relatives, both genetic and other health data can give rise to privacy and discrimination concerns among non-related individuals, as well as among the broader group of people sharing a ‘data profile’ (Barocas & Nissenbaum, 2014; Buseh et al., 2013; Mittelstadt & Floridi, 2016).

### Embodiment

In addition, post-mortem GE arises from the medical view of genetic samples as parts of our bodies that can be ‘banked’ for material: “the human body is becoming perceived as a biobank or raw material consisting of genes, cells and tissues” (Karvonen et al., 2018). Proponents of GE think that genetic biobanking is more invasive than health data collection, but we disagree. While genetic analysis does require a DNA sample taken from blood or saliva, this involves almost no physical risk. When collection takes places after death (e.g. from residual blood samples), the physical risk vanishes entirely as the deceased can no longer be hurt. Thus, similar to routine collection of observational health data, DNA collection comes with minimal risk to the health and wellbeing of the subject (Wendler & Rid, 2015). In the example of collecting DNA from residual blood samples, there is no damage to the deceased person at either level of abstraction (physical or informational) when the body remains intact and the genetic code is correctly sequenced and stored. In that case, damage to the data or sample is more likely to occur from a particular *use* in a research project (see Sect. 5).

Genetic data are also embodied^12^ in a socio-cultural sense, as we already alluded to above. Human biological material has always been viewed as an extension of people’s spiritual being (Sahota, 2014). However, human behaviour and identity result from a combination of factors, just like our health, and are not determined merely by genetics (Parens et al., 2006). This is reflected in how people think about data privacy. Evans and Burke (2008) hypothesised that “most people would feel more comfortable sharing their CYP2C9 alleles with a third party than their social security number, previous hospitalizations, or history of testing for sexually transmitted diseases”. Indeed, in one study 92% of respondents regarded their Social Security numbers as exceptionally private, while a minority of 44% thought their genetic test results required special protection (Kaufman et al., 2009). The public was similarly divided in another study where 52% supported GE (Global Alliance, 2017). In our own interviews with sudden cardiac arrest survivors, socio-economic data was regarded as more personal than DNA (Bak et al., 2021). Just like DNA sequences, other types of information can also form an extension of one’s being, although the extent to which data are embodied will differ per context.

In this regard, Floridi (2013) describes people as “informational bodies”: as well as having physical bodies, humans are living bodies whose self is constituted by information [cf. Marx’s organic/inorganic body, Husserl’s and Plessner’s Körper/Leib distinction (Butler, 2019; Krüger, 2010; Öhman & Floridi, 2017)]. Currently, the information age is blurring the distinction between analogue and digital, and informational entities obtain a higher ontological status than before (Floridi, 2013; Nettleton, 2004). For instance, artificially generated journalists and virtual social media influencers can hardly be distinguished from real persons.^13^ The same trend is seen for the virtual afterlife. While Facebook profiles could already be ‘memorialized’, i.e. turned into a shrine to mourn someone’s passing, personal data of the deceased are now being used to create personalized *chatbots* resembling an interacting self, e.g. to be used in psychotherapy (Benoit et al., 2020).^14^ Such digital clones will likely become valuable also for health research, which is increasingly conducted in partnership with large internet companies (Horn & Kerasidou, 2020). An older example is the 1994 Virtual Human Project (VHP) that virtualized the bodies of people who had donated them for research, by digitally scanning cross-sections of tissue with CT and MRI technology. The VHP is analogous to the Human Genome Project as both projects have turned the human body into an archive or a ‘digital substance’ (Waldby, 2003).

This brings us to the final aspect of embodiment used as argument for GE: the dual (legal) status of DNA samples as biological materials and as personal data.^15^ However, neither data stored in databases nor samples stored in lab freezers are themselves sources of information, but they are *resources* from which information is constructed. Genetic and other medical databases can be described as “spaces of convergence for computing and biology” (Chow-White and García-Sancho, 2012), with data functioning as a *boundary object* between the natural and the sociocultural (Meloni, 2016). Yet this artificial distinction between “analogue/carbon-based and digital/silicon-based” is simply a feature of the Level of Abstraction (LoA) used (Floridi, 2011). One’s physical and informational body are ontologically different in that they are realizations of that person at different LoA. Turner (1992) already described that the phenomenological perspective we adopt, depends on the ‘level of analysis’. One intentionally adopts a certain LoA, and questions about the nature of data are always teleological and tied to context: e.g. the organic aspects of genetic data are relevant when we discuss how to collect or store DNA samples, while the inorganic aspects are useful in discussions on anonymization.

### A Plea for Contextual Exceptionalism

We have seen that both genetic and other health data research can thwart the privacy interests of deceased patients and related living persons. Genetic information does not have exceptional characteristics per se, but the specific qualities of data relate to the context. For instance, genetic testing for hereditary cardiovascular illness is similar to an HIV test in the sense that both can predict future health problems; but they are different in that HIV testing will not produce incidental findings, while the genetic test will not result in stigmatisation by sexual partners. Because sensitivity of data is context-dependent, the idea of exceptionalism should be extended to other health data when the context so requires. Garrison et al. (2019) propose a notion of ‘genomic contextualism’ which highlights the importance of context and updates the term for the era of genomics. This may still give rise to specific policies for genetic data in certain situations, or as the authors explain: “tailored policies for genetic tests and information need not be predicated on a claim of global exceptionalism”.

We concur with the general conclusion by Garrison and co-authors but feel that their term still seems applicable only to genomics, so we instead make a plea for *contextual exceptionalism* in post-mortem health data research.^16^ Rather than thinking in terms of genetic/non-genetic or dead/living, researchers and oversight bodies ought to focus on whether the specific research project matches the expectation of respectful and just treatment of the data. Posthumous data should be treated by researchers with exceptional care if the context requires so, which might be because the data are highly relational or embodied (in Sect. 5 we suggest a list of contextual factors). One example of a sensitive context is the use of data from people in indigenous communities. Following the legal case of the Havasupai Tribe whose DNA samples were used by researchers for purposes outside of the given consent (Reardon & TallBear, 2012), communities have advocated for more control over (secondary uses of) their samples. Among such historically disadvantaged groups, there may be less willingness to participate in genetic studies than in other types of observational health research (McQuillan et al., 2003). However, the idea of contextual exceptionalism may help to see that non-genetic big data research could have negative implications for these communities as well (Cherrington et al., 2020).

## Post-Mortem Privacy in Practice

In health research with posthumous data, the ethics are complex because while such research concerns potentially sensitive data, it also carries the possibility of future benefit for living individuals. How should benefits for the living be weighed against the privacy interests of the deceased and other relevant persons in practice? We reflect on this using the real-world case of research with implanted cardiac devices (ICDs) and pacemakers removed after death, to which we apply the findings from the previous sections.

### The Case of Research with Data from Implanted Cardiac Devices

In the Netherlands there exist no data protection laws or guidelines specifically for observational research after death. Researchers are uncertain about what rules apply, which can be illustrated with a recent case from a Dutch university hospital where cardiologists had come into contact with a funeral care company that possessed a large number of pacemakers and implantable cardioverter defibrillators (ICDs). These had been removed when the deceased individuals were prepared for burial or cremation, and normally the devices are either destroyed or returned to the treating hospital. However, the personal data contained in them would be valuable for research, as one of the researchers said: *“The patient has died and I understand that many rights are lost then. My hope is that I can use the data on the ICDs and pacemakers for scientific research”*. The devices had belonged to patients from various hospitals and the researchers had no treatment relation with (most of) these patients during their lifetime. Would it indeed be acceptable, not only legally but morally, to use these data posthumously?

This is a sensitive topic because removal of implanted cardiac devices is an invasive act (removal used to be an act reserved for medical professionals but can now be done by funeral directors)^17^ and because the data give information about hereditary heart rhythm disorders which may be relevant for relatives. The data in these devices are relational and embodied, at least in the biological sense. However, the GDPR does not apply to deceased persons and the Dutch implementation law is no exception. National *lex specialis* consists of the Funeral Services Act which does not mention either implants or data processing, and the Medical Treatment Act which states that post-mortem data research may be performed without consent if the data are necessary for the study, which serves the public good, and if the deceased did not explicitly object whilst alive (Ploem, 2006). These conditions provide a starting point for decision-making on the ethical use of the post-mortem data for research.

### Reflection and Recommendations

We concur with Harbinja (2019) that post-mortem privacy should be recognised in either the GDPR (through an amendment that includes the dead in the definition of personal data) or national law, and that consent requirements should also apply to the dead. Moreover, we advise that *all* posthumous data processing for medical research is assessed by an institutional Research Ethics Committee (REC) or by a review body for observational research. This should be a case-by-case evaluation grounded in what we have called ‘contextual exceptionalism’ in order to propose tailored safeguards, rather than merely distinguish between genetic and other health data. Based on the (missing) information in our case, we propose a list of contextual factors to be used for evaluating health research that uses personal data posthumously, or for the development of ethical codes for specific research area (Box 1).

**Box 1:** Contextual factors for evaluating health research using post-mortem data

Aims and quality of the proposed research project (e.g. whether aims serve the public interest);
Whether the data are required for the purpose of the study (i.e., data minimisation);
What safeguards are in place (e.g. ICT security, or whether the data can be anonymised);
Sharing policies and details on how the data were obtained (e.g. commercial purposes);
Reasonable expectations of the next-of-kin (e.g. based on information provided to them);
What impact asking consent from next-of-kin would have on them and on study validity (e.g. in terms of emotional burden, or whether contact information is known);
The implications of the data for living next-of-kin and the broader community, with specific attention for disadvantaged and marginalised groups (i.e., relationality);
To what extent the data constitute the deceased person informationally (i.e., embodiment) and how the dignity of the informational body would be affected by this particular data use;
Wishes of the deceased person (e.g. expressed in consent for other research projects or as communicated to the next-of-kin whilst alive)

A few comments are in place. Firstly, this list is not exhaustive and more factors may be added after additional case studies. Secondly, some factors have a higher moral value than others. For instance, even if data can be anonymised without losing their relevance, this mitigates concerns about re-identification but it would still be morally wrong to use these data if patients or relatives had been told that devices would be destroyed. Thirdly, some questions are difficult to answer, e.g. researchers may not know the deceased person’s wishes. Empirical research could help: a survey study in the United States showed that most patients with an ICD wanted the data to be used for research when they die (Kirkpatrick et al., 2007).^18^ Interview studies may provide insight in the emotional burden on next-of-kin when approached for consent. Lastly, the embodiment criterion stresses that some, but definitely not all, data is constitutive of one’s identity. Less restrictive policies may be used for ‘dead’ pieces of information (e.g. a separate ECG recording is less sensitive than a full medical record), although this becomes increasingly difficult in the age of big data with increased linkage of datasets.

What would be our advice to the researchers from the case, if we presume that neither the patient, whilst alive, nor the relatives received any information on post-mortem use? Removal of the device does not itself require consent but the researchers’ lack of treatment relation, combined with the potential implications for living relatives, may create a moral duty to ask consent for data research from next-of-kin. However, study validity would be limited by non-responders, which in a grieving population may be many. Full data anonymization is not an option as researchers likely want linkage to the medical record. An acceptable middle ground would involve a letter by the funeral director about the transfer of data to researchers, also explaining the disclosure policy for individual findings, which should include an opt-out option. In the future, this information could be provided routinely by the funeral home. Yet the most autonomy-promoting option would be to inform the patient during placement of the ICD or pacemaker about post-mortem use of data for scientific research—this is the recommended route by the British Cardiovascular Society (Pitcher et al., 2016, Sect. 7.14). Consent then functions both as a way to promote respect for persons and to help researchers conduct posthumous research without uncertainty about the legal basis for data processing.

It is a question for future study if consent should expire at a certain time after death, in order to limit the potential for undesirable future uses (Ploem et al., 2021). Dynamic consent where data subjects are continuously updated and can change their preferences accordingly (Budin-Ljøsne et al., 2017), is impossible after death. Alternatively, people might select their own “data death date” or choose a trusted person (“posthumous data guardian”) to give consent in their stead (Shaw, 2019), and researchers could use AI-based tools to investigate what the likely preferences of the dead subject would have been (Biller-Andorno & Biller, 2019). These ideas require further research in terms of acceptability, impact on research, and whether guardians and AI accurately predict preferences. An open question is also whether relatives can cancel the deceased person’s wishes, similar to families overruling organ donation preferences in some countries; and further study is needed on procedures for disclosing findings to relatives as well as on the distinction between research and diagnostics, which may be narrow in case of rare disease research with data of the deceased (Fellmann et al., 2020).

## Concluding Remarks

In this paper we have argued that posthumous privacy violations, once carefully interpreted, are possible in principle and that further oversight is needed in the context of health data research after death. This would help protect data privacy and maintain public trust in research. We find that post-mortem privacy should be recognised in the GDPR or in national laws, while ‘contextual exceptionalism’ requires that all studies—i.e., not only in genetics—with data of deceased patients are evaluated on a case-by-case basis by research ethics committees. Namely, ethical questions about the use of data are always teleological and tied to context. We have proposed a list of contextual factors that can be used in such assessments, but further (case) studies may be needed to come to a comprehensive list. Future philosophical analyses could help shed light on the issue of data ‘expiry’ over time and on remaining questions around personhood and the self in relation to data-driven phenotyping. Even though one may critique the assumptions grounding Floridi’s information ethics, we find that adopting this gaze is valuable for the interdisciplinary field of digital health as it shows us that respect for deceased research subjects is important not only at the biological level, but also at the informational level. In the current age of big data, medical ethics ought to be complemented with some form of information ethics so that we care not merely for the dying, but also for the dead data subject.

## Footnotes

1 Regulation (EU) 2016/679 of the European Parliament and of the Council of 27 April 2016 on the protection of natural persons with regard to the processing of personal data and on the free movement of such data, and repealing Directive 95/46/EC (General Data Protection Regulation). Recital 27. OJ L 119/1. 2019.

2 Countries may have sector-specific regulations governing confidentiality of medical data post-mortem, but legislation varies widely within Europe and across the globe. For instance, France and Hungary have established protections for deceased persons’ data while the United Kingdom emphasises that personal data only exists in relation to living individuals (Harbinja, 2019). In the United States, the Health Insurance Portability and Accountability Act (HIPAA) protects health information for 50 years after the individual’s death.

3 Of note is that harm has been influentially defined as a “*wrongful* setback of interests” (Feinberg, 1984, emphasis added), but people can be harmed without wrongdoing, namely, in the case of (bad) luck. For instance, a patient can suffer iatrogenic harm as a result of medication side effects, without having been wronged by their prescribing physician. We therefore consider harms to be setbacks of interests.

4 Aristotle probably believed that the force of post-mortem events is weaker than of those occurring while still alive, as he was committed to the idea of *eudaimonia* or human flourishing as an objective condition determined in large part by the intellectual virtues of living persons (Belliotti, 2011, p. 2–4).

5 An illuminating example of an informational entity is the chess pawn (Floridi 2011 ). The actual pawn (i.e., the phenomenon) is merely a placeholder while the real pawn (i.e., the noumenon) is the informational object that captures the properties and behaviour of the pawn in the game of chess.

6 For readability, we have used the terms ‘data’ and ‘information’ interchangeably throughout this paper.

7 In IE this process is described as metaphysical entropy; the decay of information which constitutes an impoverishment of Being qua informational entity (Floridi, 2013).

8 Not asking consent for data use on the basis of a ‘rule of rescue’ (Porsdam Mann et al., 2016) is ethically unfounded as one data subject does not save the life of an identifiable person (Krutzinna et al., 2019a).

9 The definition of genetic data can be debated, but here we use it to mean any data derived from a person’s DNA sample, be it through whole genome sequencing (WGS) or a specific genetic test. Regarding health data, we mean any data related to health, including health determinants like socio-economic status (Prainsack, 2017).

10 Genetic samples are often awarded stronger protection as the unique genetic code is thought to make them more identifiable and difficult to anonymize (Gymrek et al., 2013). But in the age of big data, anonymization does not suffice as a safeguard for other health data either (Schneble et al., 2018).

11 Data are in a relation with the persons who create or process them (Prainsack, 2019), and ontologically, data are relational entities: a data-point can only exist because it differs from other data-points (Floridi, 2011). Here, however, relationality refers to the existence of relevant relationships *between persons*.

12 Embodiment is a complex term. Here, we ascribe to the phenomenological definition of embodiment as *the bodily aspects of human subjectivity*. The biological presence of one’s body is a precondition for their socio-cultural identity and existence, and the body is to be considered a subject of culture (Csordas, 1990).

13 Ian Urbina, “I Flirt and Tweet. Follow Me at #Socialbot”, *New York Times*, August 10, 2013 [https://www.nytimes.com/2013/08/11/sunday-review/i-flirt-and-tweet-follow-me-at-socialbot.html]. Matt Klein, “The Problematic Fakery of Lil Miquela Explained – An Exploration of Virtual Influencers and Realness”, *Forbes*, November 17, 2020 [https://www.forbes.com/sites/mattklein/2020/11/17/the-problematic-fakery-of-lil-miquela-explained-an-exploration-of-virtual-influencers-and-realness/].

14 Companies are also filing patents on technology to create such ‘reincarnation bots’, mostly to perfect their ability to create digital clones which can be used for personalising advertisements for the living. The commercial interests associated with posthumous (health) data research are outside the scope of this article, but the concept of a ‘Digital Afterlife Industry’ (DAI) has been introduced and elaborated in a paper by Öhman & Floridi (2017).

15 Some authors do not consider genetic samples to be data, as the data must first be extracted, whereas others believe that since the data is carried within the material, data protection rules should apply (Hänold et al., 2017).

16 This links closely to Nissenbaum’s terminology of privacy as contextual integrity (Nissenbaum, 2009), but we purposely framed in a manner analogous to the term genetic exceptionalism.

17 Guidelines of the national society of crematoria specify that ICDs and pacemakers need to be removed because of the explosion hazard when cremated and because of the environmental pollution when buried.

18 There are also initiatives from patient organisations to ask people to create a living will where they ask for autopsy and readout of the device after their death, in order to improve quality of care. Source: Heart Patients Netherlands [Hartpatiënten Nederland], “Call: have your pacemaker or ICD checked after your death [Oproep: laat uw pacemaker of ICD controleren na overlijden]” December 10, 2018 [https://www.hartpatienten.nl/nieuws/oproep-laat-uw-pacemaker-icd-stent-controleren-na-overlijden/].
